# Access to general health care among people with disabilities in Latin America and the Caribbean: a systematic review of quantitative research

**DOI:** 10.1016/j.lana.2024.100701

**Published:** 2024-03-08

**Authors:** Danae Rodríguez Gatta, Sara Rotenberg, Kasim Allel, Veronika Reichenberger, Lena Morgon Banks, Hannah Kuper

**Affiliations:** a International Centre of Evidence and in Disability, Department of Population Health, Faculty of Epidemiology and Population Health, London School of Hygiene and Tropical Medicine, London, United Kingdom; b Millennium Nucleus Studies on Disability and Citizenship (DISCA), Chile; c Nuffield Department of Primary Care Health Sciences, University of Oxford, Oxford, United Kingdom; d Department of Disease Control, Faculty of Infectious & Tropical Diseases, London School of Hygiene and Tropical Medicine, London, United Kingdom; e School of Government, Pontificia Universidad Católica de Chile, Santiago, Chile

**Keywords:** Systematic review, People with disabilities, Latin America, Caribbean, Access to healthcare, Health equity

## Abstract

In Latin America and the Caribbean (LAC), there are 85 million people with disabilities (PwD). They often experience barriers accessing healthcare and die, on average, 10–20 years earlier than those without disabilities. This study aimed to systematically review the quantitative literature on access to general healthcare among PwD, compared to those without disabilities, in LAC. A systematic review and narrative synthesis was conducted. We searched in EMBASE, MEDLINE, LILACS, MedCarib, PsycINFO, SciELO, CINAHL, and Web of Science. Eligible articles were peer-reviewed, published between January 2000 and April 2023, and compared healthcare access (utilization, coverage, quality, affordability) between PwD and without disabilities in LAC. The search retrieved 16,538 records and 30 studies were included, most of which had a medium or high risk of bias (n = 23; 76%). Overall, the studies indicated that PwD use healthcare services more than those without disabilities. Some evidence indicated that women with disabilities were less likely to have received cancer screening. Limited evidence showed that health services affordability and quality were lower among PwD. In LAC, PwD appear to experience health inequities, although large gaps exist in the current evidence. Harmonization of disability and health access data collection is urgently needed to address this issue.

## Introduction

Worldwide, there are 1.3 billion people with disabilities, a diverse group of persons with long lasting physical, mental, intellectual or sensory impairments who often face various barriers that restrict them from an equal participation in society.^1^^,^^2^ This number is expected to increase further in the coming decades due to population ageing and the rise of chronic diseases.^1^ People with disabilities often have greater health needs than the general population because of baseline health conditions and increased comorbidities.^1^^,^^3^ However, they also frequently lack access to essential and high-quality health services due to several system- and individual-level barriers, which further increase health inequities.^3^ Systemic barriers (ie, that arise at the level of the health system) include low availability of services, poor healthcare worker training, stigma and low physical and communicational accessibility along the healthcare journey.^1^^,^^3^, ^4^, ^5^ While transport and substantial additional living costs, as well as low autonomy and awareness of access to healthcare, are some of the barriers people with disabilities face at the individual level.^1^^,^^4^ Consequently, people with disabilities frequently have poorer health and on average die 10–20 years earlier than those without disabilities, even under circumstances that could have been avoided.^1^^,^^4^^,^^6^ This life expectancy gap is even higher among low- and middle-income countries (LMICs).^6^ This is why member states of the United Nations (UN) recently committed to disability inclusion in healthcare systems, including essential health services and public health interventions.^7^

Bright and Kuper (2018) explored English quantitative research on access to general healthcare services for people with disabilities in LMICs between 1995 and 2015.^8^ General healthcare corresponded to essential health services (eg, antenatal care, immunization, etc.), excluding specialist health services. The included articles used a wide range of disability and healthcare access outcomes and 46% of included studies had medium or high risk of bias, restricting the possibility to draw robust conclusions.^8^ Since this systematic review, further reviews have looked at the qualitative evidence,^9^ barriers to access healthcare,^10^ or access for specific types of disabilities.^11^

After Europe, the Americas have the highest prevalence of disability globally (19%)^1^ and about 85 million (15%) people have disabilities in Latin America and the Caribbean (LAC).^12^ The LAC region represents a diverse set of countries with important sub-regional socio-economic and health differences. In general, central America has the highest poverty rates, in contrast to the Southern Cone, although the entire region has consistently been characterized by inequality.^12^ In most countries of LAC, primary healthcare is delivered by public health providers, although countries differ in their organization of basic health coverage.^13^ For instance, some countries have national health systems (Belize, Brazil, Ecuador, El Salvador, Guyana, Honduras, Jamaica, Panama, Paraguay and Trinidad and Tobago), while others have contributory health coverage with multiple insurers (Bolivia, Chile, Colombia, Dominican Republic, Guatemala, Mexico, Peru, Suriname).^13^ Furthermore, most countries protect populations with low-income against out-of-pocket payments and catastrophic health spending, but rarely other vulnerable groups.^13^ Some well-known structural weaknesses in the health systems in LAC include fragmentation (both between public and private health systems, and within public healthcare), inequality in health access, financial constrains (eg, lowest health spending in Haiti, Venezuela, and Honduras), and lack of human resources and infrastructure.^14^^,^^15^

Disability can overlap with multiple vulnerabilities of other groups such as women, children, elderly, ethnic minorities, LGBTI+ people and migrants, whose representation varies widely across LAC.^12^ Yet, analysis on healthcare access with disability lens remains scarce. This review will respond to the current call of UN member states to document health inequities experienced by all people with disabilities and further build evidence on healthcare access for LAC.^7^ More than ten years have passed since the Pan American Health Organization established a regional strategy to improve disability data^5^ and, despite the efforts to overcome this statistical invisibility, robust diagnostic analyses are still needed.^12^ The COVID-19 pandemic revealed the still poor and unsystematic information about people with disabilities and healthcare.^16^ Thus, an in depth and systematic analysis will help identify the evidence available and the remaining data gaps in healthcare access (utilization, coverage, quality, and affordability of health services).^17^

The research question addressed by this review is whether people with disabilities experience inequalities in access to healthcare in Latin America and the Caribbean. The aim of this study is to systematically review the quantitative literature on access to general healthcare among persons with disabilities, compared to those without disabilities, in LAC. This systematic review will improve upon the previous review of Bright and Kuper (2018) by capturing recent evidence and trends in access to general healthcare and including high-income countries of LAC and non-English studies, which have been previously excluded from systematic reviews.^8^^,^^9^

## Methods

This systematic review followed the Preferred Reporting Items for Systematic Reviews and Meta-Analyses (PRISMA) guidelines^18^ (Supplementary Material 1) and was registered in the Prospective Register of Systematic Reviews (PROSPERO) under the following number: CRD42021235797.

### Search strategy and selection criteria

Studies were eligible if they were peer-reviewed articles of quantitative research with interventional or observational study designs (eg, cohorts, case–control, cross-sectional, etc.) carried out in Latin American and Caribbean countries, as defined by the World Bank in 2023.^19^ They must have been published since 2000 onwards and written in English, Spanish, Portuguese, French, or Dutch. Quantitative sections from mixed methods studies were considered. Qualitative studies, studies conducted outside LAC or multi-country studies that did not provide disaggregation for a country in LAC were excluded as well as editorials, commentaries, letters to the editor, systematic reviews, case reports, study protocols, conference abstracts, and grey literature.

Participants were people with disabilities of any gender and age group, including those who have long-term physical, mental, intellectual or sensory impairments which in interaction with various barriers may hinder their full and effective participation in society on an equal basis with others.^2^ Disability was defined in the study according to the United Nations Convention on the Rights of Persons with Disabilities (UNCRPD), the International Classification of Functioning, Disability and Health or the Social Model of Disability. It included people with specific conditions deemed likely to result in disability (eg, dementia, spina bifida, schizophrenia, etc., as listed in Iemmi et al., 2015)^20^ as well as disability measured through functioning or activity limitations (eg, Washington Group questions, activities of daily living). We excluded people with mild disabilities (eg, symptoms of depression alone rather than clinical diagnosis or major depressive disorder, some difficulty in one activity of daily living/functioning domain or mild cognitive difficulties).

Eligible studies had to include one of the following measures of access to healthcare: coverage, utilization, quality, and affordability of health services. This conceptualization was based on the World Health Organization's definition of universal health coverage and its progress monitoring indicators of coverage of essential health services.^17^^,^^21^ Among eligible studies, we also included the following secondary outcomes if available: adherence to health treatment or barriers to accessing healthcare. Outcomes could be measured within any type of general health services. The studies must have had a comparison group of people without disabilities and report measures of effect comparing people with and without disabilities.

Peer-reviewed published articles were searched on April 12th, 2023, through eight databases: EMBASE, MEDLINE, LILACS, MedCarib, PsycINFO, SciELO, CINAHL, and Web of Science. In addition, the reference lists of relevant systematic reviews were checked to identify potential articles. No language restrictions were applied; however, a date filter was applied to identify papers published after 2000. Comprehensive search strings were built with keywords and thesaurus and MeSH terms. Search terms were also identified in the full manuscript of other reviews of similar topics. The search was also conducted in Spanish and Portuguese, as these are the two main regional languages. An information specialist of London School of Hygiene and Tropical Medicine reviewed and approved the search strategy (Supplementary Material 2).

Two reviewers independently screened study titles, abstracts, and full text against the eligibility criteria. They then compared results and reached a consensus at each stage. A third reviewer resolved uncertainty or disagreement. Rayyan software was used for screening articles and recording decisions.^22^

### Data analysis

Two reviewers independently extracted data of studies selected and agreed on results. A third reviewer resolved any disagreement between individual judgements. From each article the following information was extracted: citation details, study location, study design, participant characteristics (sex, age group, type of disability and method of assessment), outcome measures and method of assessment, results among participants with and without disabilities, summary of results (eg, measures of effect), type of health service used, barriers to healthcare and quality measures. Data extracted were recorded in a Microsoft Excel spreadsheet.

A narrative synthesis was conducted on each type of outcome of access to healthcare. Summary of results with measures of effect (eg, prevalence ratios with 95% confidence intervals [CI]) presented as unadjusted, age-sex adjusted and/or multivariable adjusted or mean with standard deviation were collected. Results were organised in subgroups according to outcome measurements and thereafter according to type of impairment (mental, physical, sensory, intellectual, or multiple impairments). Finally, a meta-analysis was intended for synthesis of results in case of sufficient homogeneity in healthcare access outcomes and across disability-specific groups.

Included studies were independently checked against quality criteria and then assessed for risk of bias by two reviewers using an adaptation of the SIGN50 guidelines.^23^ Risk of bias was assessed through the study design, participants, outcomes and data analysis and additional criteria were available for case–control and cohort studies regarding the comparability of the groups and study design (Table 1). Any disagreement was discussed together with a third reviewer. Each study was graded as low, medium, or high risk of bias, depending on the criteria fulfilled and the possibility of altering the conclusions of the study. Studies with high risk of bias were excluded from the analysis of health outcomes.

**Table 1:** Quality assessment criteria.

## Results

**Fig. 1:**
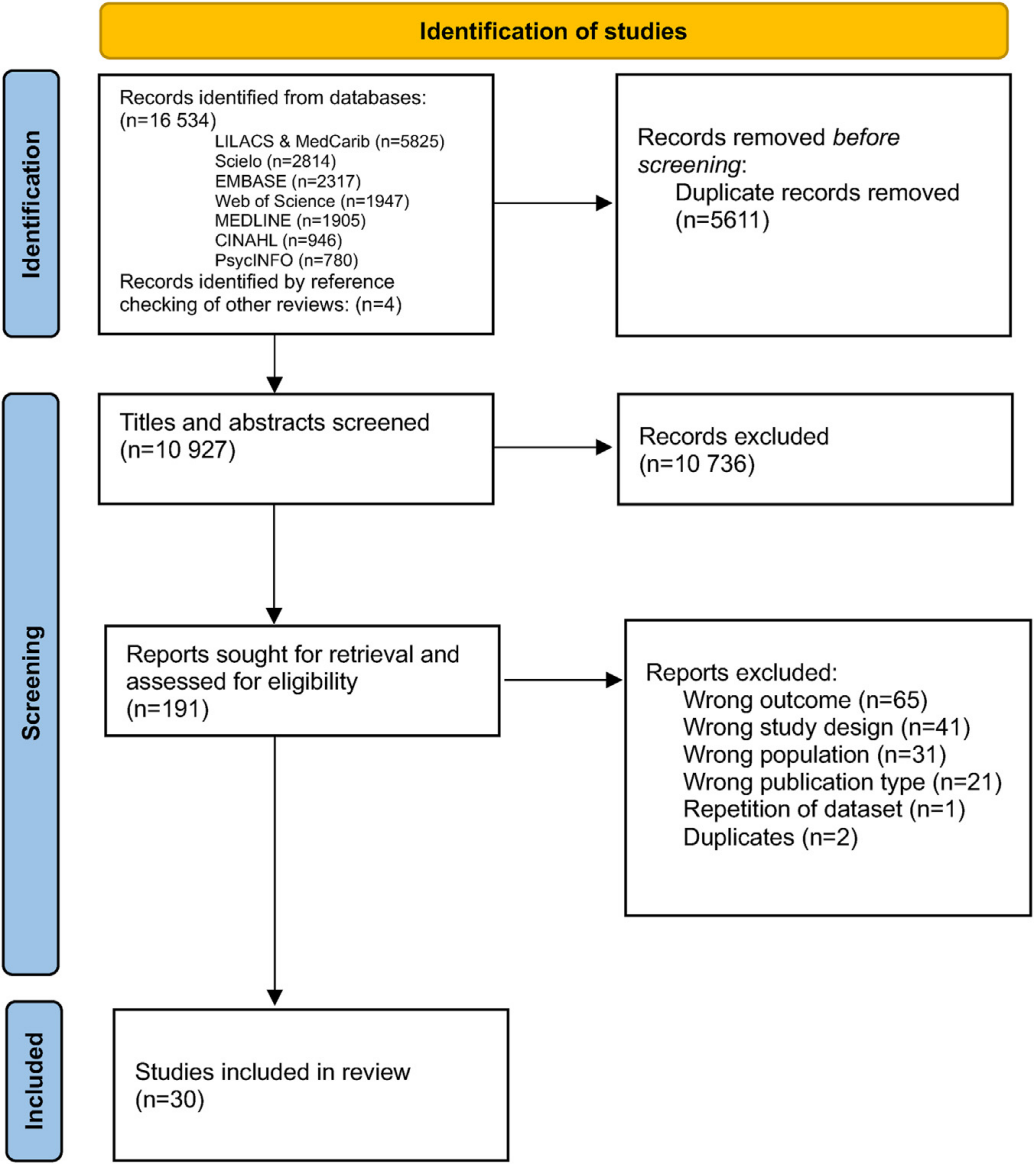
**PRISMA flow diagram of study selection and identification**.

The initial search retrieved 16,534 records. Four additional studies were found through reference checking.^24^, ^25^, ^26^, ^27^ After deduplication, the titles and abstracts of 10,927 articles were independently screened. Then, 191 articles were fully screened and finally, 30 studies were included in this systematic review (Fig. 1); of which 8 had been also previously included in Bright and Kuper's (2018) review.^8^

**Fig. 2:**
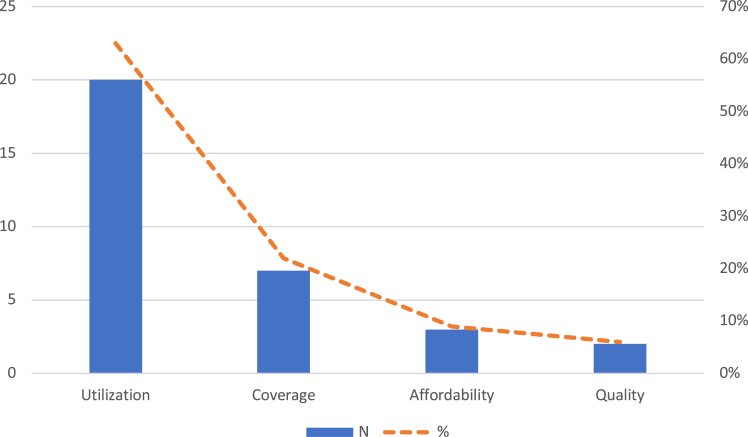
**Health access outcomes measured across included studies (n = 23)**.

Table 2 shows the main characteristics of the articles included. Most studies had a cross-sectional design (n = 24; 80%), were conducted in Brazil (n = 19; 63%) and in urban areas (n = 19; 63%). Articles were most frequently published in English language (n = 23; 77%) and from 2010 onwards (n = 27; 90%). Most participants were adults (n = 14; 47%) or of mixed age groups (n = 13; 43%). Participants often had any type of self-reported disability (n = 8; 26%) or functioning limitations (n = 8; 26%). Utilisation of healthcare was the most frequent outcome reported (n = 20; 63%) (Fig. 2). Health services often were outpatient visits (n = 16; 31%) and health treatment or medication (n = 12; 24%). The quality assessment revealed that most studies had a medium risk of bias (n = 16; 53%). Studies with high risk of bias (n = 7) were excluded from the synthesis analysis of health outcomes presented below.^24^^,^^28^, ^29^, ^30^, ^31^, ^32^, ^33^

**Table 2:** Characteristics of included studies (n = 23).

Variable	Category	N	%
Decade of publication	2000	3	10%
	2010	20	67%
	2020	7	23%
Country	Brazil	19	63%
	Chile	5	17%
	Colombia	1	3%
	Guatemala	1	3%
	Haiti	1	3%
	Mexico	1	3%
	Peru	1	3%
	Multiple	1	3%
Country income level	High income	5	17%
	Upper-middle income^a^	24	80%
	Lower-middle income	1	3%
Study location	Urban^b^	19	63%
	Urban and rural	11	37%
Study language^c^	English	23	77%
	Portuguese	4	13%
	Spanish	3	10%
Study design	Cross-sectional	24	80%
	Case-control	5	17%
	Cohort	1	3%
Disability group^d^	Any self-reported disability	8	26%
	Functional/activity limitations	8	26%
	Psychosocial disabilities	6	19%
	Hearing impairments	4	13%
	Intellectual/learning disabilities	3	10%
	Physical disabilities	2	6%
Age group	Mixed/all ages	13	43%
	Older adults ( ≥ 60 years) only	7	23%
	Adults ( ≥ 18 years) only	7	23%
	Children/adolescents only	3	10%
Outcome measured^e^	Utilization	20	63%
	Coverage	7	22%
	Affordability	3	9%
	Quality	2	6%
Type of service accessed^f^	Outpatient visits^g^	16	31%
	Health treatment/medication	12	24%
	Preventive care visits^h^	10	20%
	Hospitalization	8	16%
	Oral health services	5	10%
Risk of bias	Low	7	23%
	Medium	16	53%
	High	7	23%

a Albanese, 2011: all upper middle-income countries; expect Puerto Rico (high income) and Venezuela currently unknown (previously upper middle income).

b Albanese, 2011: four countries urban and two both urban and rural; Bernabe-Ortiz, 2016: Semi-urban.

c None of the eligible studies were found in French or Dutch language.

d There is more than one type of disability reported in Albanese, 2011.

e More than one outcome was reported in Kuper, 2018 and Fuentes-López, 2020.

f More than one type of service reported in some papers.

g Including: medical consultations, physician visits, GP appointments, home visits, emergency consultations.

h Including: antenatal care, immunization, routine check-up, PAP test, mammogram, HIV/AIDS test, prostate cancer screening.

A meta-analysis could not be performed since there was not sufficient homogeneity in the measurement of disability and healthcare access outcomes. Disability was self-reported, measured through questionnaires, clinical assessments or identified in medical or school records (Table 3). Most studies collected data under a biomedical model of disability (ie, categorised disability according to the presence of impairments or medical conditions) (n = 22; 73%). Most healthcare outcomes were collected through questionnaires and were applied during in-person interviews; only two studies collected data from patient's records within the last 12 months.^46^^,^^52^ However, healthcare outcomes were measured by different types of services and period (Table 4).

**Table 3:** Summary information of included studies by disability type (n = 30).

First author, year	Country	Study design	Source of participants	Type of disability	Description and method to assess disability	Participants		Age range	Health access measure	Risk of bias
						With disabilities, n (%)	Without disabilities, n			
Amorim, 2011^34^	Brazil	Cross-sectional	Population	Hearing or visual	Self-reported hearing or visual impairment	Hearing 141 (14%); Visual 188 (19%)	619	>50 years	Utilization	Medium
Castro, 2013^35^	Brazil	Cross-sectional	Population	Any type of disability	Self-reported disability (physical or sensory impairment; multiple disability)	492 (18%)	2198	>11 years	Utilization	Medium
Araya Vallespir, 2014^28^	Chile	Cross-sectional	Primary care clinic	Any type of disability	Self-reported disability (physical, mental, or sensory impairment)	20 households	405 households	>14 years	Quality	High
Sato, 2015^25^	Brazil	Cross-sectional	Population	Any type of disability	Self-reported health status as bedridden	36 (3%)	1305	≥60 years	Coverage	Medium
Rotarou, 2017^36^	Chile	Cross-sectional	Population	Any type of disability	Self-reported disability (physical, mental, or sensory impairment)	7459 (10%)	68,695	≥18 years	Affordability	Medium
Sakellariou, 2017^27^	Chile	Cross-sectional	Population	Any type of disability	Self-reported disability (physical, mental, or sensory impairment)	5766 (9%); 5718 (16%)	60,515; 29,576	25–65 years; 50–75 years	Coverage	Medium
Granados-Martinez, 2019^29^	Mexico	Cross-sectional	Population	Any type of disability	Self-reported disability in household (physical, mental, or sensory impairment)	Median (SD) = 7 (0.196)	Median (SD) = 93 (0.419)	≥65 years	Affordability	High
Macarevich Condessa, 2021^37^	Brazil	Cross-sectional	Population	Any type of disability	Self-reported disability (physical, intellectual, or sensory impairment)	5445 (10%)	51,756	≥18 years	Utilization	Low
Albanese, 2011^38^	Multiple^a^	Cross-sectional	Population	Functional limitation	Self-reported severe or extreme difficulty in mobility	2237 (5–30%)^b^	n/a	≥65 years	Utilization	Low
Nascimiento, 2012^39^	Brazil	Cross-sectional	Registry	Activity limitation	Activities of daily living (Katz; Lawton and Brody)	100 (16%)	519	≥60 years	Utilization	Low
Dellaroza, 2013^40^	Brazil	Cross-sectional	Population	Activity limitation	Activities of daily living (Basic and instrumental)	BADL 566 (45%); IADL 567 (45%)	705; 704	≥60 years	Utilization	Medium
Danquah, 2015^41^	Haiti	Case-control	Population	Functional limitation	Washington Group Short Set of Questions	178	178	≥5 years	Utilization	Low
Bernabe-Ortiz, 2016^26^	Peru	Case-control	Population	Functional limitation	Washington Group Short Set of Questions	161	161	≥5 years	Coverage	Medium
Kuper, 2018^42^	Guatemala	Case-control	Population	Functional limitation	Washington Group Extended Set of Questions	707	465	>2 years	Coverage, quality	Low
Montoro Pazzini Watfe, 2020^43^	Brazil	Cross-sectional	Primary care clinic	Functional limitation	World Health Organization Disability Assessment Schedule; short version	Manaus 446 (66%); Sao Paulo 396 (56%)	533	≥60 years	Utilization	Low
León-Giraldo, 2021^44^	Colombia	Cross-sectional	Population	Functional limitation	World Health Organization Disability Assessment Schedule	Mean = 4.735	n/a	All ages	Affordability	Medium
Gonçalves, 2008^45^	Brazil	Cross-sectional	Primary care clinic	Psychosocial	Psychotic, mood, substance abuse, anxiety, eating and somatoform disorders; Structured Clinical Interview	385 (51%)	369	>14 years	Utilization	Medium
Castelo, 2012^46^	Brazil	Cross-sectional	Primary care clinics	Psychosocial	Lifetime bipolar disorder with moderate/severe functional impairment; Mood Disorder Questionnaire	55 (8%)	665	18–70 years	Utilization	Low
Fujii, 2012^47^	Brazil	Cross-sectional	Population	Psychosocial	Major Depressive Disorder (PHQ-9), self-reported depression, and depression diagnosed by physician	1105 (10%)	8684	≥18 years	Utilization	Medium
García-Huidobro,2012^30^	Chile	Case-control	Registry	Psychosocial	Major Depressive Disorder; electronic clinical register	206	412	>18 years	Utilization	High
Huang, 2014^48^	Brazil	Cross-sectional	Population	Psychosocial	Major Depressive Disorder; International Classification of Diseases, Geriatric Mental State, and Neuropsychiatric Inventory	99 (5%)	1973	≥65 years	Utilization	Medium
Chiavegatto Filho, 2015^49^	Brazil	Cross-sectional	Population	Psychosocial	Major Depressive Disorder and Anxiety Disorders^c^; WMH-CIDI questionnaire	n/a	n/a	≥18 years	Utilization	Medium
Bisol, 2008^24^	Brazil	Cross-sectional	Schools	Hearing	Hearing loss; registry special school for the Deaf	42 (46%)	50	15–21 years	Coverage	High
Freire, 2009^50^	Brazil	Cross-sectional	Population	Hearing	Permanent hearing loss; audiometry	126 (10%)	1184	≥15 years	Utilization	Medium
Fuentes-López, 2020^51^	Chile	Cross-sectional	Population	Hearing	Self-reported bilateral severe-to-profound hearing loss	745	n/a	≥21 years	Utilization, coverage	Medium
Miranda, 2022^31^	Brazil	Cross-sectional	Referral centre, hospital	Hearing	Deaf children; registry care referral institution for the deaf	16	48	3–14 years	Utilization	High
Albanese,2011^38^	Multiple	Cross-sectional	Population	Intellectual	Dementia; 10/66 algorithm or DSM-IV dementia	1299 (7–12%)^d^	n/a	≥65 years	Utilization	Low
Oliveira, 2013^32^	Brazil	Case-control	Special needs centres	Intellectual	Down syndrome, cerebral palsy, autism, or intellectual disability; registry special needs school	103	103	>12 years	Utilization	High
da Silva, 2019^52^	Brazil	Cohort	Hospital	Intellectual	Severe-moderate intellectual disability; Baseline Pediatric Overall Performance Category	148 (20%)	610	1 month–16 years	Utilization	Medium
Debossan, 2022^53^	Brazil	Cross-sectional	Hospital	Physical	Rare genetic disease (Mucopolysaccharidoses and Osteogenesis Imperfecta); medical records	70	70	3–27 years	Utilization	Medium
Kessler, 2022^33^	Brazil	Cross-sectional	Population	Physical	Self-reported physical disability within household	10,878 (8%)	128,342	≥18 years	Coverage	High

Note: We reported number and percentage of participants whenever possible and calculated the total number of participants per group (ie, with or without disability) whenever studies only reported percentage. Decimals were rounded off. Abbreviations: BADL, basic activities of daily living; IADL, instrumental activities of daily living; n/a, not available; PHQ-9, Patient Health Questionnaire 9; WMH-CIDI, World Mental Health–Composite International Diagnostic Interview.

a Mexico, Peru, Cuba Dominican Republic, Puerto Rico, Venezuela.

b Cuba 546 (19%), Dominican Republic 439 (22%), Puerto Rico 603 (30%), Peru urban 143 (10%), Peru rural 30 (5%), Venezuela 204 (11%), Mexico urban 126 (13%), Mexico rural 146 (15%).

c Including: panic disorder, agoraphobia, simple phobia, social phobia, generalized anxiety disorder, obsessive compulsive disorder, post-traumatic stress disorder, and separation anxiety.

d Cuba 333 (11%), Dominican Republic 242 (12%), Puerto Rico 233 (12%), Peru urban 130 (9%), Peru rural 36 (7%), Venezuela 145 (7%), Mexico urban 93 (9%), Mexico rural 87 (9%).

**Table 4:** Summary of health access outcomes (n = 23).

First author, year	Type of disability	Description of health access measure	Health access measure among participants		Measure of effect (95% CI)/p-value	Summary direction of effect	Risk of bias
			With disabilities	Without disabilities			
**I. Utilization**							
Amorim, 2011^34^	Hearing or visual impairment	Prostate cancer screening; lifetime	Hearing impairment 30%; visual impairment 58%	43%	aPR hearing impairment = 0.93 (0.81–1.08); visual impairment = 1.10 (1.01–1.20)	Mixed^a^	Medium
Castro, 2013^35^	Any type of disability	Hospitalization; last 12 months	Visual 7%; hearing 13%; physical 33% impairment; Multiple disability 23%	6%	aPR visual = 0.85 (0.45–1.60); hearing = 1.59 (0.88–2.86); physical impairment = 3.77 (2.00–7.11); Multiple disability = 3.26 (1.62–6.55)	Mixed^a^	Medium
Macarevich Condessa, 2021^37^	Any type of disability	Dental visits; last 12 months	34%	45%	aOR = 0.74 (0.83–0.66)	Lower^a^	Low
Albanese, 2011^38^	Functional limitation	Use of community healthcare services; last 3 months	n/a	n/a	Pooled aPR 1.02 (0.96–1.09) [aPR Cuba = 0.83 (0.74–0.92); Peru urban = 1.21 (1.03–1.41)]^b^	Mixed^a^	Low
Nascimiento, 2012^39^	Activity limitation	Physician visits; last 12 months	None = 3 (7%); 1–5 = 58 (13%); ≥6 = 39 (31%)	None = 42 (93%); 1–5 = 390 (87%); ≥6 = 86 (69%)	p < 0.0001	Higher^a^	Low
		Hospitalization; last 12 months	None = 63 (12%); ≥1 = 37 (39%)	None = 461 (88%); ≥1 = 57 (61%)	p < 0.0001	Higher^a^	
Dellaroza, 2013^40^	Activity limitation	Hospitalization and >4 consultations; last 12 months	BADL 45%; IADL 45%	44%; 43%	PR BADL = 1.02 (0.76–1.36); IADL = 1.04 (0.81–1.33)	Higher	Medium
Danquah, 2015^41^	Functional limitation	Health centre visits (≥16 years); last year	0 = 34 (33%); 1–2 = 27 (26%); ≥3 = 42 (41%)	0 = 44 (42%); 1–2 = 35 (33%); ≥3 = 26 (25%)	aOR 1–2 versus 0 = 1.0 (0.5–2.0); ≥3 versus 0 = 2.1 (1.0–4.3)	Mixed	Low
		Health centre visits (<16 years); last year	0 = 40 (53%); 1–2 = 14 (19%); ≥3 = 21 (28%)	0 = 33 (45%); 1–2 = 26 (36%); ≥3 = 13 (18%)	aOR 1–2 versus 0 = 0.4 (0.2–0.9); ≥3 versus 0 = 1.3 (0.5–2.9)	Mixed	
Montoro Pazzini Watfe, 2020^43^	Functional limitation	Family physician visits; last 3 months	Sao Paulo yes = 60%, no = 53%; Manaus yes = 71%, no = 63%	Sao Paulo yes = 48%, no = 52%; Manaus yes = 42%, no = 58%	p = 0.18	Higher	Low
Gonçalves, 2008^45^	Psychosocial	GP visits; last 12 months	None = 60 (16%); 1 = 51 (13%); 2–5 = 132 (35%); 5–10 = 82 (22%); >10 = 57 (15%)	None = 104 (28%); 1 = 81 (22%); 2–5 = 111 (30%); 5–10 = 44 (12%); >10 = 26 (7%)	p = 0.02, when controlled for chronic disease	Higher^a^	Medium
		Emergency visits; last 12 months	None = 113 (30%); 1 = 90 (24%); 2–5 = 107 (28%); >5 = 67 (18%)	None = 194 (54%), 1 = 94 (26%), 2–5 = 56 (16%), >5 = 15 (4%)	p < 0.0001, when controlled for chronic disease	Higher^a^	
		Examinations	None = 86 (23%); 1 = 97 (26%); 2–5 = 132 (35%); >5 = 64 (17%)	None = 154 (40%); 1 = 111 (30%); 2–5 = 78 (21%); >5 = 32 (9%)	p = 0.002, when controlled for chronic disease	Higher^a^	
Castelo, 2012^46^	Psychosocial	≥4 GP visits; last 12 months	23 (42%)	165 (25%)	aRR = 1.92 (1.11–3.41)	Higher^a^	Low
Fujii, 2012^47^	Psychosocial	Physician visits; last 6 months	Mean (SD) = 8.4 (10.5)	Mean (SD) = 3.3 (5.6)	p < 0.05	Higher^a^	Medium
		Emergency visits; last 6 months	43%	17%	p < 0.05	Higher^a^	
		Hospitalization; last 6 months	18%	8%	p < 0.05	Higher^a^	
Huang, 2014^48^	Psychosocial	≥3 outpatient visits; last 3 months	41%	26%	adjusted Ratio of means = 1.50 (1.23–1.84)	Higher^a^	Medium
		Hospitalization; last 3 months	15%	4%	aPR = 2.87 (1.64–5.00)	Higher^a^	
Chiavegatto Filho, 2015^49^	Psychosocial	Health professional visit; last 12 months	n/a	n/a	aOR depression = 1.63 (1.14–2.33); anxiety = 1.85 (1.40–2.45)	Higher^a^	Medium
Freire, 2009^50^	Hearing impairment	Physician visits; last 2 months	55%	43%	PR = 1.3 (1.10–1.51)	Higher^a^	Medium
		Hospitalization; last 12 months	17%	8%	PR = 2.1 (1.42–3.14)	Higher^a^	
Fuentes-López, 2020^51^	Hearing impairment	GP visits	n/a	n/a	aOR = 1.78 (1.18–2.66)	Higher^a^	Medium
Albanese, 2011^38^	Intellectual	Use of community healthcare services; last 3 months	n/a	n/a	Pooled aPR 0.93 (0.90–0.97) [aPR Cuba = 0.87 (0.76–0.98); Peru rural = 1.12 (0.72–1.75)]^c^	Mixed^a^	Low
Silva, 2019^52^	Intellectual	Hospital readmissions; last 12 months	Yes = 33 (29%); No = 79 (71%)	Yes = 36 (6%); No = 574 (94%)	aOR = 1.08 (1.05–1.29)	Higher^a^	Medium
Debossan, 2022^53^	Physical	Dental visits ever	Yes = 27 (39%), No = 43 (61%)	Yes = 49 (70%), No = 21 (30%)	aOR = 0.19 (0.43–0.08)	Lower^a^	Medium
**II. Coverage**							
Sato, 2015^25^	Any type of disability	Receipt of influenza vaccination	75%	74%	PR = 1.01 (0.81–1.26)	Null	Medium
Sakellariou, 2017^27^	Any type of disability	Receipt of a Pap test (25–65 years); last 3 years	48%	63%	aOR = 0.698 (0.65–0.75)	Lower^a^	Medium
		Receipt of mammogram (50–75 years); last 3 years	46%	61%	aOR = 0.771 (0.72–0.82)	Lower^a^	
Bernabe-Ortiz, 2016^26^	Functional limitation	Sought healthcare for health problem	Always = 61%; sometimes = 26%; never = 13%	Always = 64%; sometimes = 30%; never = 6%	p = 0.20	Lower	Medium
Kuper, 2018^42^	Functional limitation	Received treatment, if have any general health condition	357 (61%)	149 (53%)	aOR = 1.4 (1.0–1.9)	Higher^a^	Low
		Sought treatment for health problem; last 12 months	254 (76%)	78 (72%)	aOR = 1.2 (0.7–2.1)	Higher	
		Sought antenatal care (15–49 years); last 5 years	n/a	n/a	aOR = 0.4 (0.1–1.0)	Lower^a^	
		Children vaccinated (5–9 years)	94%	88%	aOR = 2.6 (0.3–20.2)	Higher	
Fuentes-López, 2020^51^	Hearing impairment	No receipt of gynecological check-up; last 3 years	97%;	84%	PR = 1.2 (1.1–1.2)	Lower^a^	Medium
		No receipt of Pap test; last 3 years	65%	42%	PR = 1.6 (1.3–1.8)	Lower^a^	
		No receipt of mammogram test; last 3 years	43%	37%	PR = 1.2 (0.7–1.6)	Lower	
**III. Affordability**							
Rotarou, 2017^36^	Any type of disability	Difficulty paying for treatment due to cost	11%	5%	aOR = 1.91 (1.74–2.09)	Lower^a^	Medium
León-Giraldo, 2021^44^	Functional limitation	Catastrophic health expenditure	n/a	n/a	aOR = 1.04 (1.01–1.06)	Higher catastrophic health expenditure^a^	Medium
**IV. Quality**							
Kuper, 2018^42^	Functional limitation	General feeling of being completely disrespected	47 (9%)	13 (4%)	aOR versus “completely respected” = 1.9 (1.0–3.7)	Lower^a^	Low
		Difficult to understand information given	121 (22%)	42 (14%)	aOR versus “easy” = 1.6 (1.1–1.4)	Lower^a^	
		Difficult to be understood by health provider	106 (20%)	43 (14%)	aOR versus “easy” = 1.3 (0.8–1.9)	Lower	

Abbreviations: BADL, basic activities of daily living; GP, general practitioner; IADL, instrumental activities of daily living; n/a, not available; PAP test, Papanicolaou test.

a Strong or some evidence against a null association.

b Dominican Republic = 0.94 (0.84–1.05); Puerto Rico = 1.04 (0.99–1.09); Peru rural = 1.38 (0.97–1.96); Venezuela = 0.98 (0.89–1.09); Mexico urban = 1.10 (0.89–1.13); Mexico rural = 1.01 (0.89–1.09).

c Dominican Republic = 0.97 (0.83–1.12); Puerto Rico = 0.95 (0.89–1.02); Peru urban = 0.89 (0.72–1.09); Venezuela = 0.86 (0.73–1.00); Mexico urban = 0.92 (0.80–1.06); Mexico rural = 0.93 (0.78–1.12).

Table 4 shows the summary of outcomes measured, where 17 studies examined differences in healthcare utilization between people with and without disabilities. Nine studies (53%)–eight cross sectional studies and one cohort study–reported strong evidence of a higher utilization among people with disabilities (outpatient visits or hospitalizations).^39^^,^^45^, ^46^, ^47^, ^48^, ^49^, ^50^, ^51^, ^52^ However, two studies indicated that people with disabilities utilized oral health services less often than people without disabilities.^37^^,^^53^ Three studies (18%) found some evidence of mixed utilization levels.^34^^,^^35^^,^^38^ The studies focussed on people with hearing impairment or psychosocial disabilities all showed that they utilized health services more often than the comparison groups without disabilities.^45^, ^46^, ^47^, ^48^, ^49^, ^50^, ^51^ Studies without significant results showed a trend towards either higher (n = 2) or mixed (n = 1) utilization levels among people with disabilities.^40^^,^^41^^,^^43^

Coverage of key services was examined in five studies, and three found statistically significant differences by disability status among women. For example, women with disabilities had lower coverage of preventive health services such as cancer screening, gynaecological check-ups and antenatal care than those without disabilities.^27^^,^^42^^,^^51^ The rest of the studies indicated either no differences or lower coverage levels.^25^^,^^26^ Furthermore, the two cross-sectional studies reporting on affordability revealed that people with disabilities had more difficulties affording health services or had catastrophic health expenditures than persons or households without disabilities.^36^^,^^44^ Finally, a case–control study in Guatemala reported that the quality of healthcare services was lower among people with functional limitations than those without. They found that people with disabilities felt disrespected or found it difficult to understand the information given during a health treatment than people without disabilities.^42^

Two cross-sectional studies reported additional disaggregation by age, gender, and level of severity. Fuentes-López & Fuente (2020) found that older adults with hearing impairments were more likely to have a routine health checkup than older adults without disabilities and that women with hearing impairments visited GPs more often than those without disabilities.^51^ Macarevich Condessa et al. (2021) found people with severe disabilities utilized oral health services less often than those with milder disabilities.^37^ Only Albanese et al. (2011) disaggregated results by study location, however no clear differences were observed in the utilization of community health services among people with disabilities in urban versus rural Peru and Mexico.^38^ Finally, although some studies adjusted their analyses by ethnicity, disaggregated results by indigenous people or afro-descendants were not found among included studies.

Four studies–two case-controls^41^^,^^42^ and two cross-sectional studies^27^^,^^36^–reported barriers to access healthcare services. People with disabilities faced about 2–4 times more difficulties with the availability of health services^41^^,^^42^ and access to health facilities (age-sex-adjusted odds ratio [OR] (95% CI) = 4.4 (1.9–10.2)), than those without disabilities.^41^ They also reported difficulties in arriving at health facilities (aOR 2.95 (2.72–3.20)), being attended (aOR 1.72 (1.61–1.84)), or obtaining a doctor's appointment (aOR = 1.83 (1.72–1.94)).^36^ Women with disabilities also believed that cancer screening tests did not apply to them (26–34%) or that they did not need them (around 26%).^27^

**Fig. 3:**
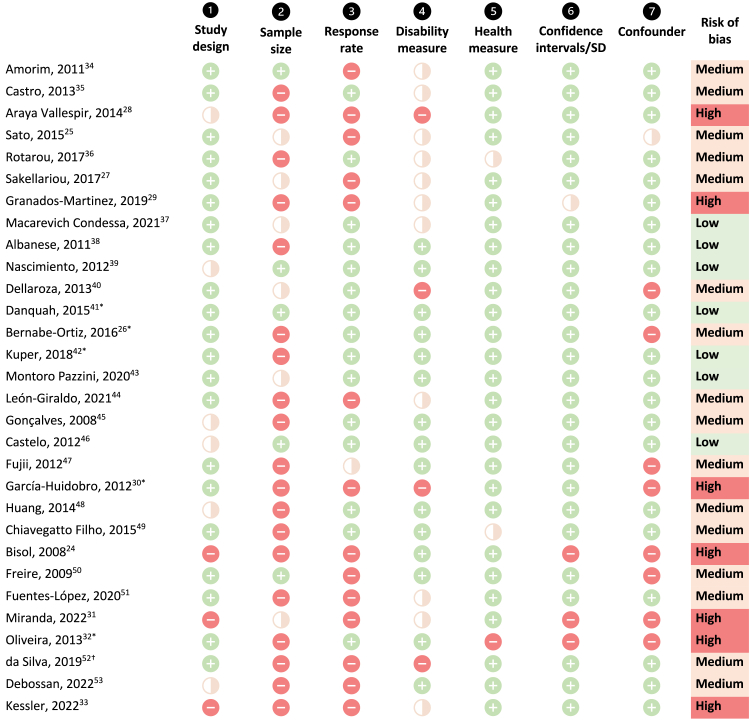
**Quality assessment and risk of bias across studies (n = 30)**.

Fig. 3 presents the risk of bias assessment for each study. Studies had low (n = 7; 23%), medium (n = 16; 53%) and high (n = 7; 23%) risk of bias (Fig. 3). Almost all studies (n = 28; 90%) presented a health access measure clearly defined in the methods section and confidence intervals or standard deviations in the results (n = 26; 87%). However, sample size calculations were often not reported in the paper or incomplete (n = 25; 83%). Similarly, response rates were often not reported (n = 14; 47%). Generally, case–control studies (n = 5) had comparable and clearly defined cases and controls.

## Discussion

This systematic review included 30 studies of quantitative evidence on general healthcare access among people with and without disabilities in Latin America and the Caribbean. Many studies indicated that people with disabilities use healthcare services more than those without disabilities. The few studies reporting on healthcare coverage had inconsistent results, although, there was some evidence that women with disabilities were less likely to have received cancer screening than those without disabilities. Both the affordability and quality of health services were reported to be lower among people with disabilities than those without. Overall, the evidence suggests that people with disabilities may experience health inequities in LAC.

Our results are consistent with other systematic reviews that found that people with disabilities more frequently use primary care services, outpatient care or are admitted to the hospital than those without disabilities.^8^^,^^11^ However, the two studies reporting on oral health services found a lower use among people with disabilities, especially among those with severe limitations, and people with rare genetic diseases.^37^^,^^53^ Furthermore, health coverage appeared to be limited for some services. Two studies found that women with disabilities have lower coverage of cancer screening than those without disabilities.^27^^,^^51^ Similar findings were reported in a meta-analysis within high-income settings, where women with disabilities were less likely to have breast (22%) or cervical (33%) cancer screening than those without disabilities.^54^ People with disabilities face barriers in accessing sexual and reproductive health services; for instance, in sub-Saharan Africa they face inaccessible physical health infrastructure, stigma and discrimination across different levels.^10^ However, only limited interventions exist to promote sexual and reproductive health among this population in LMICs.^55^ Further analyses on healthcare coverage are needed, including a wider range of preventive services (eg, family planning, HIV, immunization, chronic diseases, etc.).

Despite the finding of higher utilisation, people with disabilities might not have access to affordable or quality healthcare. Only two studies reported on affordability of healthcare. In comparison to those without disabilities, our findings suggest that people with disabilities find it difficult to afford services or face catastrophic health expenditures.^36^^,^^44^ Previous systematic reviews, also found some evidence of higher health expenditures for people with disabilities^8^^,^^56^ and a strong association between disability and poverty in LMICs.^57^ Catastrophic health expenditures and additional living costs among people with disabilities and their families might be particularly problematic in LAC, where household wages remain limited.^1^^,^^5^^,^^12^^,^^56^ Very little evidence was available on quality of healthcare. One study found that people with disabilities felt disrespected or reported that health information was difficult to understand.^42^ A meta-synthesis of qualitative evidence in LMICs highlighted that health worker attitudes and health information are common barriers faced by people with disabilities when accessing primary healthcare.^9^ Similarly, a global synthesis of qualitative evidence found that women with disabilities encounter lack of communication tools in health centres and lack of appropriate skills and training among health providers.^58^ Training of health workers is essential to improve the healthcare experience^1^ and according to a recent review, sustained learning with multiple teaching methods and participation of people with disabilities could be a successful disability training model.^59^ Additional evidence on affordability of health services is key to inform policy required on financial protection measures tailored to the LAC region. Similarly, evidence on the quality of healthcare is essential to monitor the effectiveness of the interventions, which should respond to the specific needs of people with disabilities to improve wellbeing, quality of life and participation in society.

This systematic review has some limitations that should be considered. Most studies were conducted in Brazil (n = 19; 63%); thus, findings may reflect to a large extend Brazil's context and limit the generalizability to other countries in the LAC region. Furthermore, most studies had a cross-sectional design which restricts the possibility to analyse causal paths between disability and healthcare access. Moreover, many studies (n = 25; 83%) partially presented or did not report sample size calculations and therefore, we could not assess their power and likelihood of reporting extreme results. There was a high level of heterogeneity in the measurement of disability and healthcare access, which made comparison across studies difficult. Although countries included in this review ratified the UNCRPD, most data were collected under a biomedical model of disability, despite the call for supporting both the individual and social dimension of disability.^60^ Additionally, both disability and healthcare access outcomes were often self-reported. This could imply a risk of reporting bias among participants and further limit the robustness of the evidence. We also excluded participants with mild disabilities (eg, depressive symptoms alone) and despite these being systematically excluded, we could have introduced some selection bias by trying to differentiate mild from severe disabilities. Moreover, our review did not include grey literature and might have some level of publication bias.

Although the joint analysis of all people with disabilities reinforces the issue of health equity faced by this group, disability is diverse. Health needs vary by several factors (eg, health conditions, impairment type, age, gender, environment, residence, etc.) and even throughout the lifecourse.^1^ Healthcare access among people with intellectual or learning disabilities was likely under-represented in this review. This finding supports the urgent call to improve data collection on people with intellectual and psychosocial disabilities, including in the LAC region.^12^ Similarly, other groups of people with disabilities are not represented in this analysis. For instance, people living in large institutional settings such as care homes, prisons, etc., which have been found to be often excluded from censuses and household surveys in Latin America and the Caribbean.^12^ Furthermore, disability could overlap with vulnerabilities of other minority groups (eg, indigenous people, afro-descendants, migrants, etc.) and due to lack of data, an intersectional analysis could not be conducted.^12^ Future studies should report on healthcare access among people with disabilities by gender, impairment type, residence, and intersecting identities.

Despite these limitations, we present the most comprehensive literature and analysis from a region with limited evidence available. This systematic review has important strengths. We registered a study protocol and conducted the search strategy in several languages (English, Spanish and Portuguese). We also searched for studies in multiple databases and independently assessed information. In contrast with Bright and Kuper's and other previous reviews,^8^^,^^9^ our analysis included 23% of studies in non-English language (n = 7) and 17% from high-income countries (n = 5), which would have not been included in other reviews.

In conclusion, people with disabilities appear to experience health inequities related to general healthcare access in Latin America and the Caribbean. Our findings provide some evidence that confirms the higher utilization of healthcare among people with disabilities in LAC, than those without disabilities. But important data and quality gaps exist in current research, especially in coverage, affordability, and quality of healthcare. Further harmonization of disability and health access data collection is urgently needed to assess health equity among populations with and without disability, including those with invisible disabilities. A health research agenda going forward on health equity and universal health coverage will facilitate evidence-based policy making in inclusive health for people with disabilities in Latin America and the Caribbean.

## Contributors

DRG, HK and LMB conceived the study. DRG developed the search strategy and conducted the search. DRG, HK, LMB, SR, KA, and VR conducted the first and second screenings of titles and abstracts. DRG, HK, LMB, and KA performed full text screening. DRG, SR, and KA conducted data extraction and bias evaluation. DRG wrote and revised the manuscript drafts. All authors made intellectual contributions and critically reviewed and accepted the final manuscript before its submission.

## Data sharing statement

The protocol of this systematic review can be found in PROSPERO under the following number: CRD42021235797.

## Declaration of interests

We declare no competing interests.

## Appendix A Supplementary data

Translated summary
