# Supplementary material for: Access to general health care among people with disabilities in Latin America and the Caribbean: a systematic review of quantitative research
Source: Lancet Reg Health Am. 2024 Mar 8;32:100701. doi: 10.1016/j.lana.2024.100701 (PMC10943476; doi:10.1016/j.lana.2024.100701)
Supplement: Translated summary [file mmc1.docx]

**Editor note:** *This translation in Spanish was submitted by the authors and we reproduce it as supplied. It has not been peer reviewed. Our editorial processes have only been applied to the original abstract in English, which should serve as reference for this manuscript.*

**RESUMEN**

**Introducción:** En el mundo, hay 1.300 millones de personas con discapacidad, incluidos 85 millones en América Latina y el Caribe (ALC). Las personas con discapacidad a menudo enfrentan barreras para acceder a atención de salud y mueren, en promedio, entre 10 y 20 años antes que las personas sin discapacidad. Este estudio tuvo como objetivo revisar sistemáticamente la literatura cuantitativa sobre el acceso a la atención general de salud de personas con discapacidad, en comparación con aquellas sin discapacidad, en ALC.

**Métodos:** Se realizó una revisión sistemática y síntesis narrativa siguiendo las orientaciones de PRISMA. Se realizaron búsquedas en EMBASE, MEDLINE, LILACS, MedCarib, PsycINFO, SciELO, CINAHL y Web of Science utilizando una estrategia de búsqueda exhaustiva en inglés, español y portugués. Los artículos elegibles debían ser revisados por pares, publicados entre enero de 2000 y abril de 2023, y comparar el acceso general a la atención de salud (utilización, cobertura, calidad, asequibilidad) entre personas con y sin discapacidad en ALC. Dos revisores seleccionaron los estudios de forma independiente, extrajeron los datos y evaluaron el riesgo de sesgo. Esta revisión fue prerregistrada en PROSPERO [CRD42021235797].

**Resultados:** La búsqueda arrojó 16 538 artículos y se incluyeron 30 estudios, la mayoría con riesgo de sesgo medio o alto (n=23; 76%). La mayoría de los estudios tuvieron un diseño transversal (n=24; 80%), fueron realizados en Brasil (n=19; 63%) y con adultos (n=14; 47%). El tipo de discapacidad fue con mayor frecuencia discapacidad auto informada (n=8; 26%) o limitaciones de funcionamiento (n=8; 26%). En general, los estudios incluidos indicaron que las personas con discapacidad utilizan los servicios de atención de salud más que aquellas sin discapacidad. Hubo cierta evidencia de que las mujeres con discapacidad tenían menos probabilidades de realizarse pruebas de detección del cáncer. Evidencia limitada mostró que la asequibilidad y la calidad de los servicios de salud eran menores entre las personas con discapacidad. No se disponía de datos desglosados por género o etnia.

**Interpretación:** Las personas con discapacidad parecen experimentar inequidades en salud en ALC, aunque existen grandes brechas en la evidencia actual (por ejemplo, cobertura, calidad, asequibilidad). Se necesita urgentemente armonizar la recopilación de datos sobre discapacidad y acceso a la salud para abordar este problema.

**Financiamiento:** Este estudio fue apoyado por la Agencia Nacional de Investigación y Desarrollo (ANID); Beca de Doctorado en el Extranjero Becas Chile (Beca 72210471). Hannah Kuper cuenta con el apoyo de una cátedra de investigación global del NIHR (301621); Lena Morgon Banks con la subvención PENDA de FCDO y el Arts and Humanities Council (subvención 102866EH); y Sara Rotenberg con una beca Rhodes (Rhodes Trust).
