# Supplementary material for: Access to general health care among people with disabilities in Latin America and the Caribbean: a systematic review of quantitative research
Source: Lancet Reg Health Am. 2024 Mar 8;32:100701. doi: 10.1016/j.lana.2024.100701 (PMC10943476; doi:10.1016/j.lana.2024.100701)
Supplement: Supplementary Material [file mmc2.docx]

**Supplementary material**

**Access to general health care among people with disabilities in Latin America and the Caribbean: a systematic review of quantitative research**

List of supplementary material

[PRISMA Checklist 2](#_Toc152843485)

[EMBASE peer-reviewed search strategy 4](#_Toc152843486)

# PRISMA Checklist

| **Section and Topic** | **Item #** | **Checklist item** | **Location where item is reported** |
| --- | --- | --- | --- |
| **TITLE** | | |  |
| Title | 1 | Identify the report as a systematic review. | 1 |
| **ABSTRACT** | | |  |
| Abstract | 2 | See the PRISMA 2020 for Abstracts checklist. | 2 |
| **INTRODUCTION** | | |  |
| Rationale | 3 | Describe the rationale for the review in the context of existing knowledge. | 3 |
| Objectives | 4 | Provide an explicit statement of the objective(s) or question(s) the review addresses. | 4 |
| **METHODS** | | |  |
| Eligibility criteria | 5 | Specify the inclusion and exclusion criteria for the review and how studies were grouped for the syntheses. | 4 |
| Information sources | 6 | Specify all databases, registers, websites, organisations, reference lists and other sources searched or consulted to identify studies. Specify the date when each source was last searched or consulted. | 5 |
| Search strategy | 7 | Present the full search strategies for all databases, registers and websites, including any filters and limits used. | 5 |
| Selection process | 8 | Specify the methods used to decide whether a study met the inclusion criteria of the review, including how many reviewers screened each record and each report retrieved, whether they worked independently, and if applicable, details of automation tools used in the process. | 5 |
| Data collection process | 9 | Specify the methods used to collect data from reports, including how many reviewers collected data from each report, whether they worked independently, any processes for obtaining or confirming data from study investigators, and if applicable, details of automation tools used in the process. | 5 |
| Data items | 10a | List and define all outcomes for which data were sought. Specify whether all results that were compatible with each outcome domain in each study were sought (e.g. for all measures, time points, analyses), and if not, the methods used to decide which results to collect. | 5 |
|  | 10b | List and define all other variables for which data were sought (e.g. participant and intervention characteristics, funding sources). Describe any assumptions made about any missing or unclear information. | 5 |
| Study risk of bias assessment | 11 | Specify the methods used to assess risk of bias in the included studies, including details of the tool(s) used, how many reviewers assessed each study and whether they worked independently, and if applicable, details of automation tools used in the process. | 5,6 |
| Effect measures | 12 | Specify for each outcome the effect measure(s) (e.g. risk ratio, mean difference) used in the synthesis or presentation of results. | 5 |
| Synthesis methods | 13a | Describe the processes used to decide which studies were eligible for each synthesis (e.g. tabulating the study intervention characteristics and comparing against the planned groups for each synthesis (item #5)). | 5 |
|  | 13b | Describe any methods required to prepare the data for presentation or synthesis, such as handling of missing summary statistics, or data conversions. | n/a |
|  | 13c | Describe any methods used to tabulate or visually display results of individual studies and syntheses. | 5 |
|  | 13d | Describe any methods used to synthesize results and provide a rationale for the choice(s). If meta-analysis was performed, describe the model(s), method(s) to identify the presence and extent of statistical heterogeneity, and software package(s) used. | 5 |
|  | 13e | Describe any methods used to explore possible causes of heterogeneity among study results (e.g. subgroup analysis, meta-regression). | n/a |
|  | 13f | Describe any sensitivity analyses conducted to assess robustness of the synthesized results. | n/a |
| Reporting bias assessment | 14 | Describe any methods used to assess risk of bias due to missing results in a synthesis (arising from reporting biases). | 5,6 |
| Certainty assessment | 15 | Describe any methods used to assess certainty (or confidence) in the body of evidence for an outcome. | 5 |
| **RESULTS** | | |  |
| Study selection | 16a | Describe the results of the search and selection process, from the number of records identified in the search to the number of studies included in the review, ideally using a flow diagram. | 6 |
|  | 16b | Cite studies that might appear to meet the inclusion criteria, but which were excluded, and explain why they were excluded. | 6 |
| Study characteristics | 17 | Cite each included study and present its characteristics. | 6 |
| Risk of bias in studies | 18 | Present assessments of risk of bias for each included study. | 6,7 |
| Results of individual studies | 19 | For all outcomes, present, for each study: (a) summary statistics for each group (where appropriate) and (b) an effect estimate and its precision (e.g. confidence/credible interval), ideally using structured tables or plots. | 6,7 |
| Results of syntheses | 20a | For each synthesis, briefly summarise the characteristics and risk of bias among contributing studies. | 6,7 |
|  | 20b | Present results of all statistical syntheses conducted. If meta-analysis was done, present for each the summary estimate and its precision (e.g. confidence/credible interval) and measures of statistical heterogeneity. If comparing groups, describe the direction of the effect. | 6 |
|  | 20c | Present results of all investigations of possible causes of heterogeneity among study results. | 6,7 |
|  | 20d | Present results of all sensitivity analyses conducted to assess the robustness of the synthesized results. | n/a |
| Reporting biases | 21 | Present assessments of risk of bias due to missing results (arising from reporting biases) for each synthesis assessed. | 6,7 |
| Certainty of evidence | 22 | Present assessments of certainty (or confidence) in the body of evidence for each outcome assessed. | 6 |
| **DISCUSSION** | | |  |
| Discussion | 23a | Provide a general interpretation of the results in the context of other evidence. | 8 |
|  | 23b | Discuss any limitations of the evidence included in the review. | 9 |
|  | 23c | Discuss any limitations of the review processes used. | 9 |
|  | 23d | Discuss implications of the results for practice, policy, and future research. | 9 |
| **OTHER INFORMATION** | | |  |
| Registration and protocol | 24a | Provide registration information for the review, including register name and registration number, or state that the review was not registered. | 4 |
|  | 24b | Indicate where the review protocol can be accessed, or state that a protocol was not prepared. | 4 |
|  | 24c | Describe and explain any amendments to information provided at registration or in the protocol. | n/a |
| Support | 25 | Describe sources of financial or non-financial support for the review, and the role of the funders or sponsors in the review. | 2, 6 |
| Competing interests | 26 | Declare any competing interests of review authors. | 10 |
| Availability of data, code and other materials | 27 | Report which of the following are publicly available and where they can be found: template data collection forms; data extracted from included studies; data used for all analyses; analytic code; any other materials used in the review. | 4,5 |

*From:*  Page MJ, McKenzie JE, Bossuyt PM, Boutron I, Hoffmann TC, Mulrow CD, et al. The PRISMA 2020 statement: an updated guideline for reporting systematic reviews. BMJ 2021;372:n71. doi: 10.1136/bmj.n71

For more information, visit: <http://www.prisma-statement.org/>

# EMBASE peer-reviewed search strategy

| **#** | **Searches** | **Annotations** |
| --- | --- | --- |
| 1 | ((disabilit* or disable* or handicap* or function* limitation* or function* diversit* or dependen* or special need* or rare diseas* or capacit*) adj6 (person* or people or individ* or patient* or subject* or adult* or elderly)).ti,ab. |  |
| 2 | (Physical* adj5 (impair* or deficienc* or disable* or disabili* or handicap* or incapacit*)).ti,ab. |  |
| 3 | (Cerebral pals* or Spina bifida or Muscular dystroph* or Arthriti* or Osteogenesis imperfecta or Musculoskeletal abnormalit* or Musculo-skeletal abnormalit* or Muscular abnormalit* or Skeletal abnormalit* or Limb abnormalit* or Amputation* or Clubfoot or Poliomyeliti* or Paraplegi* or Paralys* or Paralyz* or Hemiplegi* or wheelchair user* or wheel chair user*).ti,ab. |  |
| 4 | exp wheelchair user/ |  |
| 5 | ((Hearing or Acoustic or Ear or Ears) adj5 (loss* or impair* or deficienc* or disable* or disabili* or handicap*)).ti,ab. |  |
| 6 | ((Visual* or Vision or Eye or eyes) adj5 (loss* or impair* or deficienc* or disable* or disabili* or handicap*)).ti,ab. |  |
| 7 | (Deaf* or Blind*).ti,ab. |  |
| 8 | exp Hearing impairment/ |  |
| 9 | exp vision disorders/ |  |
| 10 | (Schizophreni* or Psychos#s or Psychotic Disorder* or Schizoaffective Disorder* or Schizophreniform Disorder* or Dementia* or Alzheimer* or anxiet* disorder* or depression* or Bipolar Disorder* or personality disorder*).ti,ab. |  |
| 11 | exp "schizophrenia and disorders with psychotic features"/ |  |
| 12 | exp Dementia/ or exp Alzheimer disease/ |  |
| 13 | ((Intellectual* or Mental* or Psychological* or Developmental or cognitive) adj5 (impair* or retard* or deficienc* or disable* or disabili* or handicap* or ill* or dysfunction* or deficit* or incapacit*)).ti,ab. |  |
| 14 | exp Mentally Disabled Persons/ |  |
| 15 | ((communication or language or speech or learning) adj5 (disorder* or disabilit* or impair* or deficit* or deficienc*)).ti,ab. |  |
| 16 | exp Learning Disorders/ |  |
| 17 | ((child* or juvenile or adolescent* or teenager*) adj3 (disable* or handicap* or disabili*)).ti,ab. |  |
| 18 | exp Disabled Children/ |  |
| 19 | ((genetic or hereditary or inherited or congenital) adj3 (disease* or ill* or syndrome or defect* or disorder* or condition* or malformation or anomal* or abnormalit*)).ti,ab. |  |
| 20 | exp genetic disorder/ |  |
| **21** | or/1-20 | Disability |
| 22 | (Caribbean or Latin America or Central America or South America).ti,ab. |  |
| 23 | exp Latin America/ or exp south america/ or exp central america/ or exp caribbean/ |  |
| 24 | (Guadaloupe* or Aruba* or Martinique or Martinican* or "Turks and caicos islands" or Turks Islander* or virgin island* or Peru* or Argentin* or Brazil* or Chile* or Colombia* or Venezuela* or Cayman* or Cayman island* or Puerto Ric* or Saint Barthelem* or ST Barthelemy or Guatemal* or Ecuador* or Bolivia* or Haiti* or Cuba* or Dominican Republic or Dominican* or Hondura* or Paraguay* or Nicaragua* or El Salvador or Salvador* or Costa Rica* or Panama* or Uruguay* or Jamaica* or "Trinidad and Tobago" or Trinidadian* or Tobagonian* or Guyan* or Suriname* or Belize* or Baham* or Barbad* or St Lucia* or Saint Lucia* or Grenad* or St Vincent or "Saint Vincent and the Grenadines" or Saint Vicentian* or St Vicentian* or "Antigua and Barbuda" or Antiguan* or Barbudan* or Dominica* or "Saint Kitts and Nevis" or "St Kitts and Nevis" or Kittitian* or Nevisian* or Mexic* or Curacao).ti,ab. |  |
| **25** | or/22-24 | Latin America and the Caribbean |
| 26 | ((access* or equal* or inequal* or barrier* or afford* or accept* or avail* or prevent* or treat* or diagn* or us*1 or usage or utili#ation or right* or disparit* or coverage or universal) adj3 (health or healthcare)).mp. |  |
| 27 | health care delivery/ or exp health care access/ or exp universal health care/ |  |
| 28 | exp health care utilization/ |  |
| 29 | ((clinical governance or evaluation* or qualit* or standard* or patient* need* or patient* satisfaction* or experience* or preference* or need* or satisfaction* or people-centredness or patient-centred or patient centered or attitude* or skill* or knowledge or responsiveness) adj3 (health or healthcare)).ti,ab. |  |
| 30 | exp health care quality/ |  |
| 31 | ((plan* or insurance* or program* or benefit* or expenditure* or "out-of-pocket payment*" or "financial risk protection") adj3 (health or medical)).ti,ab. |  |
| 32 | exp health insurance/ |  |
| 33 | program* acceptabilit*.ti,ab. |  |
| 34 | exp program acceptability/ |  |
| **35** | or/26-34 | Healthcare access - Universal Health Coverage |
| 36 | ((health or healthcare) adj3 (service* or agency or practice* or visiting)).ti,ab. |  |
| 37 | exp health service/ or exp health care/ |  |
| 38 | ((child* or adolescen* or p?ediatric or infant*) adj3 (service or health or healthcare or pneumoni* or lung inflamma* or pulmon* inflamma* or diarrh?ea or rehydration)).ti,ab. |  |
| 39 | exp child health care/ | UHC Tracer area: Child treatment |
| 40 | ((health or healthcare) adj3 program*).ti,ab. |  |
| 41 | exp health program/ |  |
| 42 | (palliati* adj3 (care or consultation* or medicine or therap* or surger* or treatment*)).ti,ab. |  |
| 43 | exp palliative care/ |  |
| 44 | ((primary or first) adj3 (health or care or healthcare)).ti,ab. |  |
| 45 | exp primary health care/ |  |
| 46 | (healthy people or health promotion).ti,ab. |  |
| 47 | exp health promotion/ |  |
| 48 | ((birth interval* or family planning or family building) adj3 (polic* or clinic* or service* or method*)).ti,ab. |  |
| 49 | exp family planning/ | UHC Tracer area: Family planning |
| 50 | ((matern* or obstetric* or ante natal or antenatal) adj3 (health or healthcare or control*)).ti,ab. |  |
| 51 | exp maternal health service/ |  |
| 52 | exp maternal care/ |  |
| 53 | obstetric procedure/ or exp intrapartum care/ or exp perinatal care/ or exp postnatal care/ or exp prenatal care/ or exp prepregnancy care/ | UHC Tracer area: Pregnancy and delivery care |
| 54 | ((readaptation or rehabilitation or readjustment) adj3 (functional or medical or program* or treatment* or therap*)).ti,ab. |  |
| 55 | exp rehabilitation/ |  |
| 56 | (health screening or health screening program* or population screening or mass screening).ti,ab. |  |
| 57 | exp mass screening/ |  |
| 58 | (hiv treatment* or anti-hiv agent* or anti-retroviral therap* or antiretroviral therap* or antiretroviral treatment* or ART or "anti human immunodeficiency virus agent").ti,ab. |  |
| 59 | exp anti human immunodeficiency virus agent/ | UHC Tracer area: HIV treatment |
| 60 | (malaria adj3 (eradication or prevention or prophylaxis or control)).ti,ab. |  |
| 61 | exp malaria control/ | UHC Tracer area: Malaria prevention |
| 62 | ((impregnated or insecticid* or insecticide treated or insecticide-treated) adj3 (net* or bednet*)).ti,ab. |  |
| 63 | exp insecticide treated net/ |  |
| 64 | (fluid therap* or parenteral fluid therap*).ti,ab. |  |
| 65 | exp fluid therapy/ |  |
| 66 | (diagnostic* service* or immuni#ation or immuni#ation program* or preventive service* or preventive health service* or disease prevention or "cardiovascular disease* prevention" or disease prophylaxis or preventive medication* or preventive treatment* or preventive therap* or nicotine abstin* or nicotine cessation or nicotine withdrawal or "abstinence from tobacco" or quit smoking or smoking abstin* or "tobacco use cessation" or cancer prevention or "human papillomavirus vaccine" or "human papilloma virus vaccine" or "papillomavirus vaccine*" or "papilloma virus vaccine*" or "hepatitis b vaccine*" or "hepatitis b virus vaccine*").ti,ab. |  |
| 67 | prophylaxis/ or exp cancer prevention/ or exp heart infarction prevention/ or exp immunization/ or exp smoking cessation/ | UHC Tracer area: Child immunization; Prevention of cardiovascular disease; Tobacco control |
| 68 | exp Papillomavirus Vaccines/ |  |
| 69 | exp hepatitis B vaccine/ |  |
| 70 | (sanitation or clean water or sanitary service* or toilet facilit* or hand d#sinfection or hand washing).ti,ab. | UHC Tracer area: Water and sanitation |
| 71 | exp hand disinfection/ |  |
| 72 | ((cancer or carcinoma or oncological or tumo?r) adj3 (detection or recognition or diagnos#s or cure or healing or remedy or treatment or therapy or screening)).ti,ab. | UHC Tracer area: Cancer detection and treatment |
| 73 | exp cancer diagnosis/ |  |
| 74 | exp cancer therapy/ |  |
| 75 | (tuberculosis treatment* or tuberculosis diagnos* or "anti tubercul* drug*" or antitubercul* drug* or antitubercul* agent* or tubercul* drug* or tubercul* therap* or tuberculostatic agent*).ti,ab. |  |
| 76 | exp tuberculostatic agent/ | UHC Tracer area: Tuberculosis treatment |
| 77 | ("anti hypertensi* agent*" or "anti hypertensi* drug*" or antihypertensi* agent* or antihypertensi* drug* or antihypertensi* or hypotensive agent* or hypotensive drug*).ti,ab. |  |
| 78 | exp antihypertensive agent/ |  |
| 79 | (diabetes management or "diabetes mellitus management" or "diabetes mellitus treatment" or "diabetes mellitus control" or diabetes treatment or diabetes control or "anti diabet* drug*" or antidiabet* drug* or "anti diabet* agent*" or antidiabet* agent* or antidiabetic* or hypoglyc?emic agent* or hypoglyc?emic drug*).ti,ab. |  |
| 80 | exp antidiabetic agent/ | UHC Tracer area: Management of diabetes |
| 81 | ((healthcare or service or healthcare access or diagnos* or prevention* or vaccine* or immuni#ation*) adj3 ("Human SARS coronavirus" or "SARS associated coronavirus" or SARS cov or SARS virus or SARS coronavirus or "severe acute respiratory syndrome coronavirus" or covid-19 or sars-cov-2)).ti,ab. |  |
| 82 | exp SARS coronavirus/ |  |
| 83 | ((dent* or tooth) adj3 (program* or health or healthcare or service* or treatment*)).ti,ab. |  |
| 84 | exp dental care/ |  |
| 85 | ((bed* or hospital bed*) adj3 (capacity or per capita)).ti,ab. |  |
| 86 | exp hospital bed capacity/ | UHC Tracer area: Hospital access |
| 87 | ((health worker* or healthcare worker* or "health care worker*" or health professional* healthcare professional or "health care professional" or health personnel or healthcare personnel or "health care personnel" or health practitioner or healthcare practitioner or "health care practitioner" or physician* or psychiatrist* or surgeon* or "skilled health professional*" or nurse) adj3 (density or per capita or ratio)).ti,ab. |  |
| 88 | exp nurse patient ratio/ | UHC Tracer area: Health worker density |
| 89 | (essential adj3 (medicine* or drug* or medication*)).ti,ab. |  |
| 90 | exp essential drug/ | UHC Tracer area: Access to essential medicines |
| 91 | (IHR or "international health regulation*" or WHO IHR or "IHR core capacity index" or "international health regulation* core capacity index").ti,ab. |  |
| 92 | exp international health regulation/ | UHC Tracer area: Health security |
| **93** | or/36-92 | Healthcare services |
| **94** | **35** and **93** |  |
| 95 | **21** and **25** and **94** |  |
| 96 | limit 95 to yr="2000-Current" |  |
